# Engineering online and in-person social networks to sustain physical activity: application of a conceptual model

**DOI:** 10.1186/1471-2458-13-753

**Published:** 2013-08-14

**Authors:** Liza S Rovniak, James F Sallis, Jennifer L Kraschnewski, Christopher N Sciamanna, Elizabeth J Kiser, Chester A Ray, Vernon M Chinchilli, Ding Ding, Stephen A Matthews, Melissa Bopp, Daniel R George, Melbourne F Hovell

**Affiliations:** 1 Departments of Medicine and Public Health Sciences, Penn State College of Medicine, Hershey, PA, USA; 2 Department of Family and Preventive Medicine, University of California, San Diego, San Diego, CA, USA; 3 Departments of Medicine and Public Health Sciences, Penn State Milton S. Hershey Medical Center, Hershey, PA, USA; 4 Departments of Medicine and Cellular and Molecular Physiology, Penn State College of Medicine, Hershey, PA, USA; 5 Department of Public Health Sciences, Penn State College of Medicine, Hershey, PA, USA; 6 School of Public Health, University of Sydney, Sydney, Australia; 7 Departments of Sociology, Anthropology, and Demography, Penn State University, University Park, PA, USA; 8 Department of Kinesiology, Penn State University, University Park, PA, USA; 9 Department of Humanities, Penn State College of Medicine, Hershey, PA, USA; 10 Graduate School of Public Health, San Diego State University, San Diego, CA, USA

**Keywords:** Social networks, Social environment, Social support, Built environment, Walking, Exercise, Accelerometers, Social media, Internet, Sustainability

## Abstract

**Background:**

High rates of physical inactivity compromise the health status of populations globally. Social networks have been shown to influence physical activity (PA), but little is known about how best to engineer social networks to sustain PA. To improve procedures for building networks that shape PA as a normative behavior, there is a need for more specific hypotheses about how social variables influence PA. There is also a need to integrate concepts from network science with ecological concepts that often guide the design of in-person and electronically-mediated interventions. Therefore, this paper: (1) proposes a conceptual model that integrates principles from network science and ecology across in-person and electronically-mediated intervention modes; and (2) illustrates the application of this model to the design and evaluation of a social network intervention for PA.

**Methods/Design:**

A conceptual model for engineering social networks was developed based on a scoping literature review of modifiable social influences on PA. The model guided the design of a cluster randomized controlled trial in which 308 sedentary adults were randomly assigned to three groups: WalkLink+: prompted and provided feedback on participants’ online and in-person social-network interactions to expand networks for PA, plus provided evidence-based online walking program and weekly walking tips; WalkLink: evidence-based online walking program and weekly tips only; Minimal Treatment Control: weekly tips only. The effects of these treatment conditions were assessed at baseline, post-program, and 6-month follow-up. The primary outcome was accelerometer-measured PA. Secondary outcomes included objectively-measured aerobic fitness, body mass index, waist circumference, blood pressure, and neighborhood walkability; and self-reported measures of the physical environment, social network environment, and social network interactions. The differential effects of the three treatment conditions on primary and secondary outcomes will be analyzed using general linear modeling (GLM), or generalized linear modeling if the assumptions for GLM cannot be met.

**Discussion:**

Results will contribute to greater understanding of how to conceptualize and implement social networks to support long-term PA. Establishing social networks for PA across multiple life settings could contribute to cultural norms that sustain active living.

**Trial registration:**

ClinicalTrials.gov NCT01142804

## Background

Social networks, or people with specific patterns of contact among them, have been shown to influence physical activity (PA) in epidemiological and intervention research across diverse populations [1-4]. Despite the potential importance of social networks for sustaining PA, most adults report receiving minimal social support for PA from their social networks [5-8]. Furthermore, few interventions report success in building new social networks of active individuals, or modifying the activity-promoting behaviors of existing network members [9-15]. A lack of social networks to shape PA as a normative behavior may help explain why physical inactivity is now a global pandemic and the fourth leading cause of premature death [16]. Improving our understanding of how to engineer social networks might help to increase and sustain active living.

According to principles of the scientific method, improving procedures to build social networks to sustain PA requires developing and testing hypotheses, or models, about how specific social variables influence PA [17]. A hypothesis-driven approach can improve the fidelity, or procedural integrity, of implementing and measuring social variables, provide a roadmap that delineates which social variables were tested and untested in relation to PA outcomes, and facilitate efforts to refine hypotheses and interventions. Although existing PA interventions have targeted diverse social settings, there has been limited effort to define and measure independent, mediating (process), and moderating (effect-modifier) social variables that may affect PA within those settings. Advancing our understanding of how to build social networks to sustain PA requires more specific hypotheses and models about *how* social variables influence PA.

Key social network variables that have been shown to influence PA include the structure of people’s social connections with others (e.g., number and spatial arrangement of connections) [18-20], and the functions, or action-based consequences of those connections (e.g., modeling, praise) [21-23]. Social network structure and functions are often shaped by antecedent conditions in the physical and virtual (electronically-mediated) environments, and by a community’s demographic, biological, and psychological characteristics. The physical environment (e.g., access to PA facilities, mixed-used development) can provide opportunities for social interaction and modeling related to PA across multiple contexts [24-26]. Similarly, the virtual environment can reach people in diverse settings with opportunities for PA, as about 85% of US adults have Internet access, and 69% of those adults use online social networks [27,28]. Finally, people’s demographic, biological, and psychological characteristics may determine the extent to which they seek and obtain social interactions and reinforcement, and influence, or are influenced by, the PA behavior of others [29-31]. While many existing conceptual models have included subsets of variables related to social network antecedents, structure, or functions [32-42], none of these models have integrated these variables.

Existing conceptual models have also provided limited attention to virtual interactions that occur through online networks and other electronically-mediated communication modes, despite the success of online networks such as Facebook in engaging over 1 billion users, or one-seventh of the global population [43]. Incorporating virtual interaction modes such as online networks into conceptual models and interventions for PA could complement in-person efforts to build social networks for PA. Online networks can link people to PA opportunities and help coordinate such opportunities, expand the reach of a single comment about PA, highlight previously unknown similarities among individuals, enable modeling and normative comparisons of PA, and provide instantaneous feedback and reinforcement [44]. In-person activities resulting from online interactions may stimulate online interactions that further promote PA.

Online interactions could exert different effects on PA than in-person interactions because online contacts may have limited prior acquaintance, and lack non-verbal behavioral cues. These features of online interactions might either complement or undermine PA engagement. Because online and in-person interaction modes may exert unique effects that could be targeted in interventions, both should be included in conceptual models. Including both interaction modes is consistent with multi-level ecological models which propose that increasing cues and reinforcers for PA across diverse contexts could help sustain PA [33-35].

Despite the potential for online and in-person network-building procedures to sustain PA, it is unclear if these procedures could incrementally contribute to improving outcomes of evidence-based PA interventions. To build stronger PA interventions, it is important to determine if network-building procedures improve effects beyond those achieved with existing, state-of-the-art practices. Among existing evaluations of network-building procedures for PA [9,11,45-47], only one study to our knowledge assessed the incremental contribution of online networking procedures to a prior evidence-based PA intervention [11]. Self-reported outcomes suggested that online networking procedures did not enhance intervention effectiveness. To our knowledge, no prior studies have combined online networking with a comprehensive protocol to shape in-person social interactions for PA. Given the potential additive and synergistic effects of targeting both online and in-person interactions, evaluating a more comprehensive, multi-level approach is warranted.

Therefore, this paper aims to expand knowledge about how to engineer social networks to sustain PA by: (1) outlining a conceptual model of hypothesized relations among social network variables and PA; (2) demonstrating how this model was applied to design a randomized trial that evaluated the effects of online and in-person procedures to build social networks for PA; and (3) discussing how this model may enhance PA interventions.

### Conceptual model of social networks and PA

**Figure 1:**
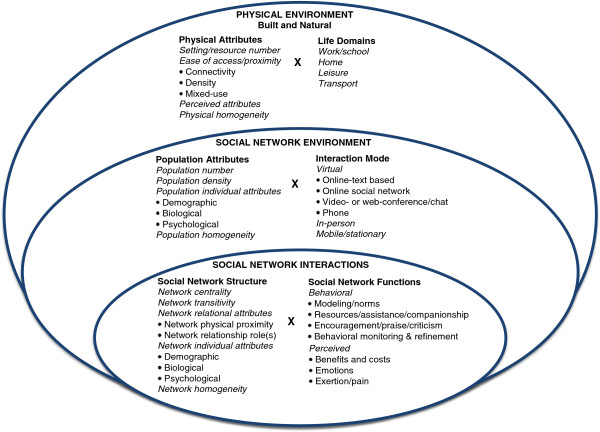
Social networks for activity promotion (SNAP) model.

The Social Networks for Activity Promotion (SNAP) model was designed to emphasize modifiable and measurable variables that could be manipulated by interventions and policies to influence social networks for PA (Figure 1). The model was developed based on a scoping review of epidemiological, intervention, and qualitative research on ecological, social network, and systems-science influences on PA and sustainable behavior change. A scoping review is appropriate when a primary goal is to integrate research on a broad topic area, and to formulate hypotheses or models to address research gaps [48].

The SNAP model is built on a multi-level ecological framework, based on the principle that multi-level interventions are more effective for sustaining behavior change [33,49]. Embedding an improved social network model in the multi-level framework ensures the social networking component is considered within the context of other levels of influence. The model proposes that PA is shaped and sustained by interactions between the *Physical Environment*, *Social Network Environment*, and *Social Network Interactions*. The physical environment can provide settings or resources that facilitate or inhibit social interactions related to PA. The social network environment provides or constrains access to: (a) populations with specific demographic, biological, and psychological attributes, and (b) modes (in-person, virtual) of interacting with such populations. The number and quality of social network interactions reflect opportunities and limits in the physical and the social network environment. Social network interactions can be defined by their structure—the attributes, spatial arrangement, and relationships among people [18-20], and by their functions—the different types of verbal and non-verbal behavior resulting from their structure that prompt and reinforce PA [21-23].

While ecological models typically define individual attributes as a separate level that interacts with other levels of the social and physical environment [33,49], network models typically define individual attributes as an integral part of the structure of social networks, or the social environment [18-20]. Consistent with network approaches, our model conceptualizes individual attributes as a component of social network structure. Consistent with ecological approaches, our model proposes that individual attributes interact with network functions, and with social network and physical environments to influence PA.

### Pathways through which concepts in SNAP model influence PA

The variables in the SNAP model may vary on their presence/absence, number, type (form or function), frequency, schedule, intensity, duration, and setting/context. Understanding the effects of these procedural variations, independently and in combination, could improve procedures for building more sustainable social networks for PA. The variables in the SNAP model may also be simultaneously operating for sedentary behaviors that compete with PA (e.g., TV watching, driving). Therefore, PA interventions and policies will need to progressively refine the design of SNAP model procedures to ensure they are powerful enough to compete with sedentary-behavior influences. Although there are many “unknowns” about how best to operationalize SNAP model variables to engineer social networks for PA, we summarize evidence from existing research that guided the hypotheses tested in this study.

Across multiple *life domains*, *physical attributes* of *built* (man-made) *and natural environments* may influence *population attributes* of people who engage in PA in these environments. For instance, neighborhood walkability, which is characterized by multiple transportation links between destinations (*connectivity*), a high concentration of destinations in a region (*density*), and *mixed-use* (e.g., recreational, occupational, residential) [50], may be especially important for increasing transport-related PA among those with weak psychosocial predispositions for PA, older adults, and low-income populations [51-53]. Similarly, park proximity and *perceived attributes* such as neighborhood greenness/aesthetics may facilitate walking among families with children [54].

Attributes of built and natural environments, together with population attributes, may also influence the extent to which people engage in PA-related social interactions through in-person and virtual (electronically-mediated) *interaction modes*. In small towns, and in economically-deprived or high-crime areas, there may be fewer physical facilities, or less variety of facilities (*physical homogeneity*) for PA than in large cities, which may reduce the range of in-person and virtual interaction opportunities for PA [55-57]. *Population individual attributes* (e.g., age, physical disability), may further influence preferences for, and engagement in, specific types of in-person or virtual interaction modes for PA [58,59]. The degree to which available PA interaction modes match the needs of specific individuals may partly reflect the extent to which these individuals are demographically and biologically similar to other population members (*population homogeneity*) [60]*.*

*Social network centrality* for PA is the number of people a person is connected to (i.e., knows or interacts with) in relation to PA. Centrality refers to a person’s position in the network (near center vs. near edge) and reflects the extent to which a person’s contacts are well-connected to others. Those with more connections tend to occupy a more central position in the network, which facilitates the ease of accessing and communicating PA-related information and opportunities [2,18,19]. Physical environments with attributes that promote PA (e.g., walkable neighborhoods, walking paths) should facilitate network centrality by fostering interaction opportunities related to PA (e.g., in-person/online walking groups, incidental social interactions while walking) [34]. These in-person and online interaction modes could enhance network centrality for PA by strengthening existing social connections and facilitating new connections [61-63]. Population attributes across different in-person and virtual interaction modes may also influence network centrality. People are more likely to form social connections with people who are similar to them, so physical settings populated by those who share common individual attributes are likely to facilitate more social connections [18,64,65]. More needs to be learned, however, about which types of attribute similarity are most salient for enhancing social connections.

While network centrality refers to the number of social contacts, *network transitivity* is the extent to which a person’s contacts know and interact with each other. Transitivity can be characterized by the quantity and duration of social interactions. Networks with high transitivity can help entrench social norms for PA or for competing sedentary lifestyles [66,67]. More frequent exposure to physical settings and interaction modes populated by people who share similar attributes (e.g., religious or professional affiliations), may increase transitivity.

The population attributes of the broader community of people available to become members of social networks, and the interaction modes they use to communicate with others, can influence *network individual* and *relational attributes*—the attributes of people who eventually become members of specific social networks for PA [64,68,69]. For instance, among individual attributes, a person surrounded by people who are low-income, or members of specific age groups, is more likely to have social connections with such individuals. Among relational attributes (i.e., attributes reflecting the joint status of two or more people), people’s frequency of interacting with diverse groups across multiple interaction modes may influence their degree of *physical proximity* to other network members, and their access to people providing different *relationship roles* (e.g., coworker, friend). Those who participate in a greater number and variety of interaction modes may have more opportunities to establish PA-related connections with people who are both similar (*network homogeneity*) and dissimilar to them [44].

People’s physical environment, social network environment, and social network structure may influence the extent to which they will be exposed to, and perform, *social network functions*, or verbal and non-verbal behaviors that can function to prompt or reinforce PA [18,19,70]. Social functions may include modeling and behavioral norms for active lifestyles, praise and encouragement for PA, providing tangible resources, assistance, or companionship to promote PA, and monitoring and refining PA relative to measurable standards [21-23]. Those with greater exposure to physical environments and interaction modes that prompt PA, and those with greater network centrality and transitivity are likely to have increased exposure to social functions that prompt and reinforce PA [18,19,70]. More extensive contact with people who serve diverse relationship roles (e.g., spouse, friend), and who have specific attributes (e.g., higher education, same gender) may also increase exposure to social functions [71,72]. Physical proximity appears to increase exposure to social functions such as companionship, but may be less important for functions such as transmitting norms [71-73]. The extent to which people receive and perform social functions has been shown to predict PA [8,70,74].

Overall, the SNAP model is consistent with existing ecological models, which propose that having multiple prompts and reinforcers for PA across diverse settings will increase PA [33-35]. It extends existing models by proposing that multiple interaction modes, in addition to multiple physical settings, can contribute to multi-level approaches to PA promotion. Interaction modes contribute to multi-level approaches by providing additional virtual settings for PA promotion, and by facilitating social interactions both within, and across, in-person and virtual settings. The model also assumes that structural and functional aspects of social networks can occur both in-person and in virtual modes, and among interconnected individuals or organized groups. Finally, the model integrates social networks with the built and natural environment, and suggests that our physical environment defines limits and opportunities that determine the existence and quality of our interactions with others.

## Methods/Design

Project WalkLink is a cluster-randomized controlled intervention trial that was designed as a preliminary test of the SNAP model (Figure 1). This section provides an overview of the WalkLink trial and illustrates how the SNAP model was applied to design study hypotheses, and intervention and measurement protocols. The WalkLink study measured all variables in the SNAP model, except for the concepts of network transitivity, network individual attributes, and network homogeneity, as assessing participants’ social contacts exceeded the scope of the study.

### Aims

Project WalkLink aimed to evaluate the relative effectiveness of three 12-week programs for engineering social networks to sustain regular walking and PA. Sedentary adults (N = 308) were randomly assigned to three groups: WalkLink+: prompted and provided feedback on participants’ online and in-person social-network interactions to expand networks for PA, plus provided evidence-based online walking program and weekly walking tips; WalkLink: evidence-based online walking program and weekly tips only; Minimal Treatment Control: weekly tips only. The primary outcome was accelerometer-measured PA. Secondary outcomes included objectively-measured aerobic fitness, body mass index, waist circumference, blood pressure, and neighborhood walkability; and self-report measures of the physical environment, social network environment, and social network interactions. Intervention fidelity was also measured. Assessments were conducted at baseline, post-program, and 6-month follow-up.

The primary specific aims of this study were to:

1. Evaluate the differential effectiveness of the WalkLink+, WalkLink, and Minimal Treatment Control groups on change in primary and secondary outcomes from baseline to post-program and 6-month follow-up.

2. Evaluate if the physical environment, social network environment, and social network interactions mediate the effect of treatment condition on PA.

3. Evaluate if the physical environment, social network environment, and social network interactions moderate the effect of treatment condition on PA.

### Hypotheses

Based on the SNAP model (Figure 1), average effect sizes for walking programs [14,75], and prior research on social mediators and moderators of PA [24,25,70], we hypothesized that:

1. Both the WalkLink+ and WalkLink groups would increase PA more than the Minimal Treatment control group.

2. The WalkLink+ group would increase PA more than the WalkLink group.

Across all treatment groups:

3. Participants exposed to a greater number and range of built and natural environment settings/resources for PA, population attributes, and interaction modes, would have greater network centrality for PA.

4. Greater network centrality for PA would increase exposure to social network functions for PA (modeling, resources/assistance/companionship, encouragement/praise, and behavioral monitoring and refinement).

5. Participants with specific relational attributes (physical proximity to PA partners, PA partners serving diverse relationship roles) would have greater exposure to social network functions for PA.

6. Participants with greater exposure to social network functions would have greater PA.

7. Change in PA would be *mediated* by the degree of change in the physical environment, the social network environment, and social network interactions.

8. Change in PA would be *moderated* by the degree of change in the physical environment, the social network environment, and social network interactions.

### Participants

The study was conducted in three separate cohorts of sedentary adults recruited from small cities and towns within the Harrisburg-Carlisle metropolitan statistical area in Pennsylvania. Conducting three independent replications of study procedures will help clarify if effects are attributable to treatment condition, or to random or systematic error. Cohort 1 (n = 64), was recruited from selected neighborhoods in the city of Harrisburg (pop 49,528); cohort 2 (n = 119) was recruited from selected neighborhoods in the city of Lebanon (pop 25,477); and cohort 3 (n = 125) was recruited from selected towns in the Harrisburg-West Shore region (combined pop 22,788). Within each cohort, participants were required to live in adjacent census tracts over an area not exceeding a 10-minute automobile drive from its two furthermost borders. This requirement was instituted to facilitate in-person social interactions among participants randomized to the WalkLink+ group, based on findings suggesting that geographic proximity facilitates in-person social interactions [73,76].

To recruit participants for all three cohorts, a list of households in each of the recruitment areas with at least one person within the study’s age range was generated from a commercial marketing database (InfoUSA). Study information was sent to the identified households via a commercial mailing service in official university envelopes. Recruitment materials directed participants to an informational website with an online screening form.

**Figure 2:**
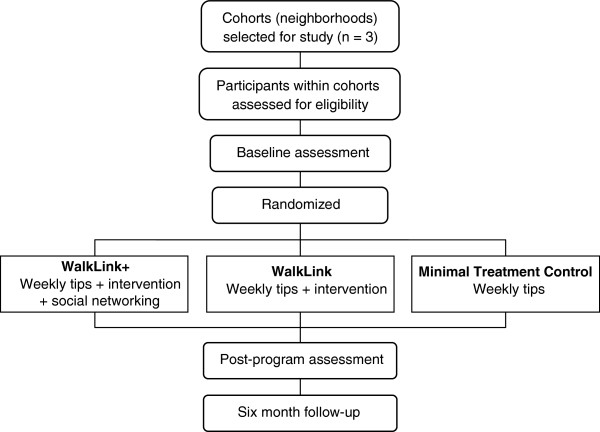
Study flow chart.

Participant inclusion criteria were: (1) physically inactive, i.e., less than 150 minutes/week of moderate intensity PA, or less than 60 minutes/week of vigorous PA; (2) aged 35–64; (3) able to speak English; (4) able to provide informed consent; (5) able to participate in moderate intensity PA. Exclusion criteria were: (1) no access to home or private work computer with Internet access; (2) body mass index greater than 39.9; (3) systolic blood pressure > 160 mm Hg or diastolic blood pressure > 100 mm Hg; (4) bone, joint or foot problems interfering with walking; (5) diabetes, pulmonary, or cardiovascular disease; (6) consume 5 or more drinks of alcohol/day; (7) pregnant; (8) not residing within targeted recruitment areas or planning to relocate during the study period. Participants with more than one medical risk factor (e.g., high cholesterol, hypertension) were required to obtain a physician’s medical approval prior to study enrollment. The study was approved by the Institutional Review Board at Pennsylvania State University College of Medicine. Figure 2 summarizes the study design.

### Randomization

At baseline, participants who indicated knowing other participants were clustered into a single randomization unit, consistent with established procedures for minimizing treatment contamination [77]. Participants were subsequently grouped into 12 strata by: (a) acquaintance with other participants/gender (three levels: cluster of acquainted participants, unacquainted males, unacquainted females); (b) age (two levels: above and below sample median age); and (c) aerobic fitness (two levels: above and below sample median fitness level). Stratification prior to randomization is recommended for cluster randomized trials to balance key variables among study conditions [77]. After randomly sequencing each participant/cluster in each stratum, computer-generated permuted block randomization (block size of three with equal allocation) was used to randomize participants to the three study conditions.

### Intervention

Table 1 illustrates the integration of intervention procedures with the SNAP model. Participants in all three conditions received a 12-week program that promoted walking and PA. All participants were encouraged to walk three or more times per week for at least 30 minutes per session, and to complete at least one other PA mode one or more times per week to achieve the benefits of cross-training. All participants also received emailed “tips of the week” once weekly, which emphasized actions to build social and environmental support for walking. Prior research suggests that this type of information has short-term, small effects on PA [78].

**Table 1:** Integration of theoretical concepts and intervention procedures

**Theoretical Concepts**	**Intervention Procedures**^**1**^	**Minimal Treatment Control**	**WalkLink**	**WalkLink+**
**Social Functions**	**Educational materials**	X	X	X
• Encouragement/prompt	-Receive same PA prescription.			
	-Receive walking “tips of the week” once weekly.			
**Social Functions**	**Shape walking quantity and speed**		X	X
• Modeling	-Receive one-on-one in-person meeting. Program activities demonstrated and practiced with corrective feedback.			
• Resources/assistance	-Given pedometer, stopwatch, walking log, and program manual.			
• Behavioral monitoring	-Prompted once weekly to submit walking logs to research staff.			
	-Self-monitor walking quantity and speed using walking log, pedometer, and stopwatch.			
• Behavioral norms	-Walking quantity and speed compared to other program participants and past performance.			
• Behavioral refinement	-Given tailored weekly goals and graphical feedback to increase walking quantity and speed.			
• Encouragement, praise				
**Environment and Social Structure and Functions**	**Online WalkLink social network site**			X
• Setting/resource access/proximity, number, homogeneity	**-**Promoted as a local program and participants informed they were living in same neighborhood as other participants.			
• Network physical proximity	-Prompted to attend four “meet the group” walks led by project staff in central neighborhood locations; participants asked to introduce themselves/talk to each participant.			
• Virtual and in-person interaction modes	-Access to WalkLink Facebook site during, and at least 1 year after program. Site activities include: posting profile, inviting family/friends/coworkers to join site, posting and joining local walking and PA events,discussion board, status updates, posting photos, “friending” other participants; received emails from site.			
• Network centrality and transitivity				
• Modeling/norms				
• Encouragement, praise				
• Resources, assistance, companionship	-Eligible for entry into drawings for gift cards contingent on posting or joining walking/activity events on WalkLink site.			
• Behavioral monitoring				
**Environment and Social Structure and Functions**	**Shape social network-building activities across in-person settings and online WalkLink site**			X
• Behavioral monitoring	-Self-monitor number of walks taken with others, and in-person community-based and Facebook-based social networking activities completed each week using checklist (Figure 3); submit networking activities once weekly to research staff.			
• Setting/resource number, homogeneity				
• Virtual and in-person interaction modes				
• Network centrality and transitivity				
• Behavioral norms	-Participation in social networking activities compared to other program participants and past performance.			
• Behavioral refinement	-Given tailored weekly goals and graphical feedback to increase social networking activities.			
• Encouragement, praise				

^1^In Cohort 1, Ning Networks was used in the place of Facebook, and no incentives were given for online social networking. All other procedures were identical across all three cohorts.

In the *Minimal Treatment Control* condition, aside from receiving the emailed tips, there was no contact between project staff and participants for 12 weeks.

The *WalkLink* condition received an introductory skill-building meeting, a program manual, and 12 weeks of tailored email coaching (Table 1). To improve walking quantity and speed, they were asked to use a program-provided Yamax SW-200 pedometer, wrist stopwatch, and walking log, and a free site (http://www.mappedometer.com/) to measure walking mileage. Participants were prompted by email each week to submit forms detailing their walking frequency, pedometer steps, duration, mileage, rating of perceived exertion, and emotional state while walking to project staff, using previously tested procedures [14]. Within one day of receipt of walking logs, participants received emailed graphical feedback that showed how their walking steps and speed compared both to their prior performance and to other participants. The feedback also provided tailored walking goals for the forthcoming week. Feedback was not automated but was generated using algorithms.

The *WalkLink+* condition received all procedures described for the WalkLink group. However, their introductory meeting also provided instruction on using a private online social networking site. In cohort 1, we used Ning Networks for the site, while in cohorts 2 and 3, based on participant feedback, we used a private Facebook site. Following the introductory meeting, four “meet the group” walks in participants’ neighborhoods were led by program staff in the first two weeks of the program to facilitate meeting other WalkLink+ members. Participants were then asked to post self-led walking or PA events on the online networking site and to join other members’ events. In cohorts 2 and 3, but not cohort 1, all participants who posted or joined events were eligible for entry into a drawing for a $25 gift card every two weeks. Participants were encouraged to invite friends and family members to join the online networking site and walking/PA events.

**Figure 3:**
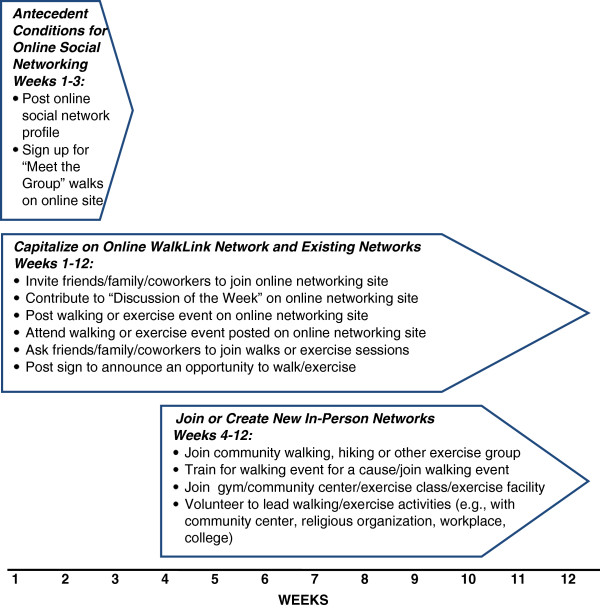
Social network-building activities for WalkLink+ group.

Participants in the WalkLink+ group were asked to self-monitor and report the number of people they walked with each week, and the number of social networking actions taken in the prior week using a checklist (Figure 3). Consistent with shaping procedures [79], we first emphasized easier networking behaviors (e.g., attending the “meet the group” walk), followed by more complex behaviors such as engaging one’s local community in walking. WalkLink+ participants received weekly graphical feedback on their number of walking partners and social networking actions relative to their past performance and other program participants, and tailored goals to increase networking activity.

An online discussion board on the online networking site presented a weekly topic related to social networking goals (e.g., leading walking groups) to enable participants to learn from others’ experiences and develop collaborative plans. Participants could also contribute their own discussion topics. Aside from posting discussion topics, program staff contributed to the online networking site only to address participant questions. A separate networking site was used for each cohort to permit tailoring to neighborhood-specific resources (e.g., local walking routes and fitness classes), and to facilitate social interactions among members living in close geographical proximity.

### Measures

Table 2 illustrates the integration of our measurement protocol with the SNAP model. Participants received $20, $30, and $40, respectively, for attending the baseline, post-program, and 6-month follow-up assessments at a clinical research center. All measurement staff received training in conducting assessments to ensure consistency to the study protocol.

**Table 2:** Integration of theoretical concepts and measurement procedures

**Theoretical Concepts**	**Measures**	**Pre**	**During**	**Post**	**6-month follow-up**
**Built and Natural Physical Environment**	**Walkability (connectivity, density, mixed-use), Perceived Attributes**				
	• Geographical Information Systems (ArcGIS 10): 0.5 mile road network buffer for each geocoded address; walkability index based on street connectivity, residential density, and land use mix [80,81]	X			
	• Walkscore [82]	X			
	• Neighborhood Environment Walkability Scale [83]	X		X	X
	**PA Settings and Resources: Number, Attributes, Homogeneity**				
	• Convenient PA facilities questionnaire [84]	X		X	X
	• Home exercise equipment questionnaire [84]	X		X	X
	• Settings where PA was done (adapted scale) [85]	X		X	X
**Population Attributes**	**Population Number and Density**				
	• Census data	X			
	**Population Individual Attributes**				
	• Background characteristics questionnaire	X			
	**Population Homogeneity**				
	• Degree of similarity on selected characteristics between each participant and other participants by each recruitment cohort [86]	X			
**Interaction Mode**	**Use of Interaction Modes for Walking and Physical Activity**				
	• Self-reported use of different communication modes (online, phone, in-person) to organize walking and PA*	X		X	X
	• WalkLink+ group only: objective monitoring of frequency and types of activities conducted (e.g., posting walks, contributing to discussions) on online WalkLink site		X	X	X
			
	**Access and Use of Interaction Modes in General**				
	• Self-reported ownership and use of cell phones to receive email*	X			
	• Self-reported use of Facebook, Twitter, and MySpace*			X	
**Social Network Centrality**	**In-Person Connections**				
	• Self-reported number of different companions for walking and PA*	X		X	X
	• Self-reported number of friends/family members participating in this program*	X		X	
	**Online Connections**				
	• WalkLink+ Group only: objective monitoring of number of “friends” on online WalkLink site		X	X	X
**Social Network Relational Attributes**	**Physical Proximity**				
	• Self-reported geographical proximity to existing walking and PA companions*	X		X	X
	**Network Relationship Roles**				
	• Self-reported types of relationships (e.g., friend, family member) with existing walking and PA companions*	X		X	X
**Social Network Functions: Behavioral**	**Resources, Assistance, Companionship, Encouragement, Praise**				
	• Social support for walking and PA scales [87]	X		X	X
	• Participant actions to prompt walking and PA scale*	X		X	X
	**Modeling/Norms**				
	• Modeling and social norms for walking and PA scale*	X		X	X
	**Behavioral Monitoring and Refinement**				
	• Goal setting and planning for walking scales (adapted) [74]	X		X	X
**Social Network Functions: Perceived**	**Physiological Exertion**				
	• Borg rating of perceived exertion immediately after each walk [88]		X		
	**Emotional States/Enjoyment**				
	• Rating of overall feeling/emotions immediately after each walk [14]		X		
	• Walking enjoyment (adapted scale) [89]	X		X	X
	**Perceived Benefits and Costs**				
	• Proximal outcome expectations (adapted scale)* [14,74]	X		X	X
**Physical Activity**	**Sedentary Behavior, and Light, Moderate, and Vigorous PA**				
	• Actigraph GT3X triaxial accelerometer, worn for 7 days, data stored as 1-min averages [90,91]	X		X	X
	**Walking Activity**				
	• Pedometer (Yamax SW-200), stopwatch, and weekly walking logs reporting walking steps and speed [92,93]		X		
	• National Health Interview Survey (2 items) [94]	X		X	X
	**PA in Different Life Domains, Sedentary Behavior**				
	• International PA Questionnaire, long version [95]	X		X	X
	**PA Modes (different types of PA)**				
	• Aerobics Center Longitudinal PA Questionnaire [96]	X		X	X
**Health Outcomes**	**Aerobic Fitness**				
	• Submaximal treadmill test: submaximal heart rate (measured by Polar heart rate monitor) during test [97]	X		X	X
	• Submaximal treadmill test: estimated VO_2_max [97]	X		X	X
	**Vitals**				
	• Pulse: calibrated hospital-grade Welch Allyn device [98]	X		X	X
	• Blood pressure: calibrated hospital-grade Welch Allyn device [98]	X		X	X
	**Body Composition**				
	• Body mass index: Physician’s balance beam scale (model Detecto 439) and calibrated Seca 242 digital stadiometer [97]	X		X	X
	• Waist circumference: Gulick II tape measure [97]	X		X	X
	**Sleep Quantity and Quality**				
	• Pittsburgh Sleep Quality Index [99]	X		X	X
	• Exposure to ambient noise during sleep scale*	X		X	X
**Intervention Fidelity**	**Participation**[100]				
	• Return rate for weekly walking logs		X		
	• Attendance rate at program-organized walks for WalkLink+ group		X		
	• Activities on online social networking site for WalkLink+ group		X	X	X
	**Intervention Receipt and Satisfaction**[100]				
	• Program evaluation survey: qualitative and quantitative assessment*			X	
	**Change in Theoretical Mediators**				
	• Degree to which theoretical mediators (listed above) changed in hypothesized direction for each of the intervention groups	X	X	X	X
	**Intervention Delivery and Quality Control**				
	• Program records, weekly meetings, staff training, walking feedback double-checked each week and 10% of feedback reviewed by PI, double-verification of manually entered data, participants recontacted for missing data, multiple sources of contact information obtained for each participant		X		

*New investigator-designed measure.

### Statistical power and sample size

The study was powered to compare the WalkLink+ and the WalkLink group relative to the minimal treatment control group on the primary outcome: mean change in accelerometer-measured PA from baseline to post-program and 6 month follow-up. Based on effect sizes from similar walking programs (i.e., programs including self-monitoring and regular feedback), we estimated an effect size (Cohen’s *d*) of *d* = .60 for both the WalkLink+ and WalkLink groups relative to the minimal treatment control group [14,101,102]. For a secondary comparison of the WalkLink+ group to the WalkLink group on primary outcomes, we estimated an effect size of *d* = .45 [14,103]. Assuming a total initial sample size of 308 (~103 per group), 20% attrition at 6-month follow-up, a two-sided test, and an overall significance level of 0.05, we estimated at least 90% power to detect group differences on the primary outcome between (1) the WalkLink+ group and minimal treatment group; and (2) the WalkLink group and minimal treatment group. Similarly, we estimated at least 80% power for the secondary comparisons of group differences between the WalkLink+ and the WalkLink groups.

### Planned statistical analyses

We will use general linear modeling (GLM), or generalized linear modeling if the assumptions for GLM cannot be met, to compare the mean change in the primary outcome (accelerometer-measured PA) and secondary outcomes (e.g., aerobic fitness, blood pressure) from baseline to post-program and six-month follow-up across the three treatment conditions (WalkLink+, WalkLink, Minimal Treatment Control). A range of covariates will be adjusted for including cohort (neighborhood), randomization strata, and key demographic characteristics. Intraclass correlation coefficients will be calculated to determine whether clustering within cohort or randomization strata require statistical adjustment. In secondary statistical analyses, we will investigate whether mediating and moderating variables (e.g., physical environment, social network environment, and social network interactions) influence change in primary and secondary outcomes. We will use SAS (Version 9.3) PROC GLM for GLM and PROC GENMOD for generalized linear modeling. When intraclass correlation coefficients are not negligible, we will use PROC GLMMIX to account for individual clustering within cohort or randomization strata.

## Discussion

Despite growing recognition that social networks both shape and mirror community-level PA, little is known about how best to engineer social networks to sustain PA. Some research based on network science suggests that modifying network structure (e.g., number and spatial arrangement of social connections) could alter population-level PA [19,104]. Other research based on ecological models suggests that modifications to social network structure cannot occur in isolation. Rather, new and modified networks are formed, changed, and sustained by physical and social influences, and by individual factors [33-35].

We proposed a conceptual model (SNAP) that integrates aspects of network science with ecological science. We also demonstrated how this model could be applied to guide intervention design and measurement. The SNAP model proposes that establishing social networks to sustain PA requires: (1) placing people in new virtual or in-person environments with people who prompt and reinforce PA; or (2) optimizing the functioning of existing social networks through increasing actions that may prompt or reinforce PA, including modeling, tangible assistance, praise and encouragement, and behavioral monitoring. Therefore, interventions or policies that target change in physical or virtual environments, or in the behaviors of people within those environments, may help build networks to sustain PA.

While the SNAP model remains to be tested and refined, it provides an initial roadmap for engineering social networks based on prior theory and empirical research. The model emphasizes modifiable independent and mediating variables that can be manipulated in PA interventions or policies, or included in multi-level analyses, and evaluated for their effects on dependent variables. Although developed to guide PA interventions, the model may also help guide selection of variables to alter social networks in other areas of health behavior change. The model extends prior research on social networks by integrating findings from network science within an ecological framework, and by proposing that multi-level ecological approaches should include both virtual and in-person modes for PA promotion across multiple settings. Having cues and prompts for PA across multiple interaction modes could increase the capacity of PA interventions to change social mediators (e.g., modeling, social reinforcement) associated with sustained PA [33,34].

Although the SNAP model’s hypotheses are based on current research and theory, there are still many “unknowns” about how to engineer social networks for PA. To address these unknowns, the model should be tested in PA interventions or epidemiological studies, with results used to refine the model and improve interventions. Model testing can evaluate how PA is influenced by manipulating the presence/absence, number, type (form or function), frequency, schedule, intensity, duration, and setting/context of variables identified in Figure 1 and Table 2. Examining effects of mediating and moderating variables across diverse geographic and demographic characteristics could help identify generalizable principles that could improve procedures to build networks for PA, as well as subgroups that might benefit from tailored interventions. Because variables can have additive, synergistic, or antagonistic effects, examining effects of different combinations of model variables might improve intervention parsimony and cost-effectiveness.

While the WalkLink study demonstrates the application of SNAP model concepts at the individual and community-level, these concepts could also be implemented and tested for effectiveness at the policy-level. Policies requiring health-behavior change programs to use more diverse interaction modes across multiple physical settings could expand population access to social cues and reinforcers for PA. Policies might also provide economic incentives to organizations that connect people to free or low-cost community-resources for PA, track PA using automated devices, or provide other related social functions. Exploring the effects of policy change on expanding access to in-person and virtual social networks for PA remains an important area for further inquiry.

Future research may also benefit from exploring areas of conceptual overlap among SNAP model variables, and developing unique operationalizable definitions of variables that minimize conceptual overlap. For instance, at least part of the concept of “network transitivity”—the extent to which network members know and interact with each other, is captured by the concept of “social functions”—the frequency of social interactions to prompt and reinforce PA. While measures of network transitivity may provide a structural representation of such interactions, it is unclear if such measures would explain additional variance in PA beyond the “social functions” variable. Similarly, the concept of “social capital” has been defined as behavioral norms [105], and access to people who can provide assistance or resources [19]. Because the concept of social capital overlaps with the concept of “social functions,” we did not include social capital in the model as a separate variable. More grant funding is needed to support team-science to develop common terminology that can be used by the growing field of social network researchers. This effort should be transdisciplinary, as scientists specializing in different disciplines are most likely to have the expertise to identify conceptual discriminations that could impact public health [106]. Establishing common terminology could improve scientific communication, and accelerate progress in developing more powerful interventions and policies.

Christakis and Fowler wrote, “a social network is like a commonly owned forest: we all stand to benefit from it, but we also must work together to ensure it remains healthy and productive” [44, p. 31]. To-date, relatively few PA interventions or policies have included comprehensive procedures to target social networks that could sustain PA. In part, the lack of emphasis on social networks may reflect the complexity and range of variables in this transdisciplinary field, and the lack of testable models and hypotheses. The present research aimed to address this gap by proposing a conceptual model with modifiable variables that could be targeted to alter social networks. The hypotheses proposed in our model require further verification. However, testing and refining these hypotheses is warranted, as changing the PA of even one person in online or in-person networks could alter PA among that person’s friends, coworkers, and family members. By gradually increasing the PA of people in social networks, we may ultimately be able to harness social networks to sustain PA.

## Abbreviations

PA: Physical activity; SNAP: Social networks for activity promotion; GLM: General linear modeling

## Competing interests

The authors declare that they have no competing interests.

## Authors’ contributions

LR led the design of the study’s conceptual model, intervention procedures, and measures, led the writing of the manuscript, and is supervising the study. JS collaborated on the design of the study’s conceptual model, intervention procedures, and measures. JK and CS collaborated on the design of the study’s intervention procedures and measures. EK is the study coordinator and contributed to the design of the study’s intervention procedures. CR supervised the design and administration of the study’s fitness testing procedures, and contributed to the study design. VC and DD contributed to the design of the study’s data analysis procedures. SM supervised the design of the study’s geographical information systems assessments. MB and DG contributed to the conceptualization of the overall study. MH contributed to the design of the study’s conceptual model, intervention procedures, and measures. All authors contributed to manuscript writing. All authors read and approved the final manuscript.

## Pre-publication history

The pre-publication history for this paper can be accessed here:

http://www.biomedcentral.com/1471-2458/13/753/prepub

## References

[B1] Yu G Renton A Schmidt E Tobi P Bertotti M Watts P Lais S A multilevel analysis of the association between social networks and support on leisure time physical activity: evidence from 40 disadvantaged areas in London Health Place 2011 17 1023 1029 10.1016/j.healthplace.2011.07.002 21784693 PMC5066841

[B2] Shelton RC McNeill LH Puleo E Wolin KY Emmons KM Bennett GG The association between social factors and physical activity among low-income adults living in public housing Am J Public Health 2011 101 2102 2110 10.2105/AJPH.2010.196030 21330588 PMC3193546

[B3] Estabrooks PA Bradshaw M Dzewaltowski DA Smith-Ray RL Determining the impact of walk Kansas: applying a team-building approach to community physical activity promotion Ann Behav Med 2008 36 1 12 10.1007/s12160-008-9040-0 18607666

[B4] Leahey TM Crane MM Pinto AM Weinberg B Kumar R Wing RR Effect of teammates on changes in physical activity in a statewide campaign Prev Med 2010 51 45 49 10.1016/j.ypmed.2010.04.004 20394768 PMC2885551

[B5] Kegler MC Swan DW Alcantara I Wrensford L Glanz K Environmental influences on physical activity in rural adults: the relative contributions of home, church and work settings J Phys Act Health 2012 9 996 1003 21975641 10.1123/jpah.9.7.996

[B6] Kiernan M Moore SD Schoffman DE Lee K King AC Taylor CB Kiernan NE Perri MG Social support for healthy behaviors: scale psychometrics and prediction of weight loss among women in a behavioral program Obesity (Silver Spring) 2012 20 756 764 10.1038/oby.2011.293 21996661 PMC4718570

[B7] Albright CL Steffen AD Novotny R Nigg CR Wilkens LR Saiki K Yamada P Hedemark B Maddock JE Dunn AL Brown WJ Baseline results from Hawaii’s Na Mikimiki project: a physical activity intervention tailored to multiethnic postpartum women Women Health 2012 52 265 291 10.1080/03630242.2012.662935 22533900 PMC3379789

[B8] Rovniak LS Sallis JF Saelens BE Frank LD Marshall SJ Norman GJ Conway TL Cain KL Hovell MF Adults’ physical activity patterns across life domains: cluster analysis with replication Health Psychol 2010 29 496 505 20836604 10.1037/a0020428 PMC3021982

[B9] Napolitano MA Hayes S Bennett GG Ives A Foster GD Using facebook and text messaging to deliver a weight loss program to college students Obesity (Silver Spring) 2013 21 25 31 10.1002/oby.20232 23505165

[B10] Baruth M Wilcox S Blair S Hooker S Hussey J Saunders R Psychosocial mediators of a faith-based physical activity intervention: implications and lessons learned from null findings Health Educ Res 2010 25 645 655 10.1093/her/cyq007 20147429

[B11] Richardson CR Buis LR Janney AW Goodrich DE Sen A Hess ML Mehari KS Fortlage LA Resnick PJ Zikmund-Fisher BJ An online community improves adherence in an internet-mediated walking program. Part 1: results of a randomized controlled trial J Med Internet Res 2010 12 e71 10.2196/jmir.1338 21169160 PMC3056526

[B12] Calfas KJ Sallis JF Oldenburg B Ffrench M Mediators of change in physical activity following an intervention in primary care: PACE Prev Med 1997 26 297 304 10.1006/pmed.1997.0141 9144753

[B13] Calfas KJ Sallis JF Nichols JF Sarkin JA Johnson MF Caparosa S Thompson S Gehrman CA Alcaraz JE Project GRAD: two-year outcomes of a randomized controlled physical activity intervention among young adults. Graduate ready for activity daily Am J Prev Med 2000 18 28 37 10.1016/S0749-3797(99)00117-8 10808980

[B14] Rovniak LS Hovell MF Wojcik JR Winett RA Martinez-Donate AP Enhancing theoretical fidelity: an e-mail-based walking program demonstration Am J Health Promot 2005 20 85 95 10.4278/0890-1171-20.2.85 16295700

[B15] Ayala GX Effects of a promotor-based intervention to promote physical activity: familias Sanas y Activas Am J Public Health 2011 101 2261 2268 10.2105/AJPH.2011.300273 22021294 PMC3222415

[B16] Kohl HW 3rd Craig CL Lambert EV Inoue S Alkandari JR Leetongin G Kahlmeier S The pandemic of physical inactivity: global action for public health Lancet 2012 380 294 305 10.1016/S0140-6736(12)60898-8 22818941

[B17] Brown CW Ghiselli EE Scientific Method in Psychology 1955 New York, NY: McGraw-Hill

[B18] Smith KP Christakis NA Social networks and health Annu Rev Sociol 2008 34 405 429 10.1146/annurev.soc.34.040507.134601

[B19] Borgatti SP Mehra A Brass DJ Labianca G Network analysis in the social sciences Science 2009 323 892 895 10.1126/science.1165821 19213908

[B20] Newman MEJ The structure and function of complex networks Siam Rev 2003 45 167 256 10.1137/S003614450342480

[B21] Heaney CA Israel BA Glanz K, Rimer BK, Viswanath K Social networks and social support Health Behavior and Health Education: Theory, Research, and Practice 2008 4 San Francisco, CA: Jossey-Bass 189 210

[B22] Berkman LF Assessing the physical health-effects of social networks and social support Annu Rev Publ Health 1984 5 413 432 10.1146/annurev.pu.05.050184.002213 6372817

[B23] Gottlieb BH Bergen AE Social support concepts and measures J Psychosom Res 2010 69 511 520 10.1016/j.jpsychores.2009.10.001 20955871

[B24] Martinez SM Ayala GX Patrick K Arredondo EM Roesch S Elder J Associated pathways between neighborhood environment, community resource factors, and leisure-time physical activity among Mexican-American adults in San Diego, California Am J Health Promot 2012 26 281 288 10.4278/ajhp.100722-QUAN-249 22548422 PMC3904444

[B25] Kegler MC Swan DW Alcantara I Feldman L Glanz K The influence of rural home and neighborhood environments on healthy eating, physical activity, and weight Prev Sci 2013 10.1007/s11121-012-0349-3 23408285

[B26] Krieger J Rabkin J Sharify D Song L High point walking for health: creating built and social environments that support walking in a public housing community Am J Public Health 2009 99 Suppl 3 S593 599 19890163 10.2105/AJPH.2009.164384 PMC2774172

[B27] Coming and Going on Facebook http://www.pewinternet.org/~/media//Files/Reports/2013/PIP_Coming_and_going_on_facebook.pdf

[B28] Demographics of Social Media Users http://www.pewinternet.org/~/media//Files/Reports/2013/PIP_SocialMediaUsers.pdf

[B29] Centola D An experimental study of homophily in the adoption of health behavior Science 2011 334 1269 1272 10.1126/science.1207055 22144624

[B30] Ashrafi E Montazeri A Mousavi M Vaez-Mahdavi MR Asadi-Lari M Influence of sociodemographic features and general health on social capital: findings from a large population-based survey in Tehran, Iran (Urban-HEART) Public Health 2012 126 796 803 10.1016/j.puhe.2012.06.013 22910445

[B31] Watts DJ Dodds PS Influentials, networks, and public opinion formation J Consumer Res 2007 34 441 458 10.1086/518527

[B32] Glass TA McAtee MJ Behavioral science at the crossroads in public health: extending horizons, envisioning the future Soc Sci Med 2006 62 1650 1671 10.1016/j.socscimed.2005.08.044 16198467

[B33] Sallis JF Cervero RB Ascher W Henderson KA Kraft MK Kerr J An ecological approach to creating active living communities Annu Rev Public Health 2006 27 297 322 10.1146/annurev.publhealth.27.021405.102100 16533119

[B34] Hovell MF Wahlgren DR Adams MA Di Clemente RJ, Crosby R, Kegler M The logical and empirical basis for the behavioral ecological model Emerging Theories and Models in Health Promotion Practice and Research 2009 2 San Francisco: Jossey-Bass 415 449

[B35] Richard L Gauvin L Raine K Ecological models revisited: their uses and evolution in health promotion over two decades Annu Rev Public Health 2011 32 307 326 10.1146/annurev-publhealth-031210-101141 21219155

[B36] Vrazel J Saunders RP Wilcox S An overview and proposed framework of social-environmental influences on the physical-activity behavior of women Am J Health Promot 2008 23 2 12 10.4278/ajhp.06070999 18785368

[B37] Sorensen G Emmons K Hunt MK Barbeau E Goldman R Peterson K Kuntz K Stoddard A Berkman L Model for incorporating social context in health behavior interventions: applications for cancer prevention for working-class, multiethnic populations Prev Med 2003 37 188 197 10.1016/S0091-7435(03)00111-7 12914824

[B38] Levasseur M Richard L Gauvin L Raymond E Inventory and analysis of definitions of social participation found in the aging literature: proposed taxonomy of social activities Soc Sci Med 2010 71 2141 2149 10.1016/j.socscimed.2010.09.041 21044812 PMC3597625

[B39] Tanford S Penrod S Social-influence model - a formal integration of research on majority and minority influence processes Psychol Bull 1984 95 189 225

[B40] Cialdini RB Goldstein NJ Social influence: compliance and conformity Annu Rev Public Health 2004 55 591 621 10.1146/annurev.psych.55.090902.142015 14744228

[B41] Berkman LF Glass T Brissette I Seeman TE From social integration to health: Durkheim in the new millennium Soc Sci Med 2000 51 843 857 10.1016/S0277-9536(00)00065-4 10972429

[B42] Blumstein DT Ebensperger LA Hayes LD Vasquez RA Ahern TH Burger JR Dolezal AG Dosmann A Gonzalez-Mariscal G Harris BN Herrera EA Lacey EA Mateo J McGraw LA Olazabal D Ramenofsky M Rubenstein DR Sakhai SA Saltzman W Sainz-Borgo C Soto-Gamboa M Stewart ML Wey TW Wingfield JC Young LJ Toward an integrative understanding of social behavior: new models and new opportunities Front Behav Neurosci 2010 34 1 9 10.3389/fnbeh.2010.00034 PMC2907235 20661457

[B43] Facebook Reports First Quarter 2013 Results http://investor.fb.com/releasedetail.cfm?ReleaseID=761090

[B44] Christakis NA Fowler JH Connected: How Your Friends’ Friends’ Friends Affect Everything You Feel, Think, and Do 2009 New York, NY: Little, Brown, & Co.

[B45] Cavallo DN Tate DF Ries AV Brown JD DeVellis RF Ammerman AS A social media-based physical activity intervention: a randomized controlled trial Am J Prev Med 2012 43 527 532 10.1016/j.amepre.2012.07.019 23079176 PMC3479432

[B46] Valle CG Tate DF Mayer DK Allicock M Cai J A randomized trial of a facebook-based physical activity intervention for young adult cancer survivors J Cancer Surviv 2013 10.1007/s11764-013-0279-5 PMC3737370 23532799

[B47] Turner-McGrievy G Tate D Tweets, apps, and pods: results of the 6-month mobile pounds off digitally (Mobile POD) randomized weight-loss intervention among adults J Med Internet Res 2011 13 e120 10.2196/jmir.1841 22186428 PMC3278106

[B48] Arksey H O’Malley L Scoping studies: towards a methodological framework Int J Soc Res Methodol 2005 8 19 32 10.1080/1364557032000119616

[B49] Sallis JF Owen N Fisher EB Glanz K, Rimer BK, Viswanath K Ecological models of health behavior Health Behavior and Health Education: Theory, Research, and Practice 2008 4 San Francisco: Jossey-Bass 465 486

[B50] Frank LD Engelke P Multiple impacts of the built environment on public health: walkable places and the exposure to air pollution Int Regional Sci Rev 2005 28 193 216 10.1177/0160017604273853

[B51] Ding D Sallis JF Conway TL Saelens BE Frank LD Cain KL Slymen DJ Interactive effects of built environment and psychosocial attributes on physical activity: a test of ecological models Ann Behav Med 2012 44 365 374 10.1007/s12160-012-9394-1 22899301

[B52] Berke EM Koepsell TD Moudon AV Hoskins RE Larson EB Association of the built environment with physical activity and obesity in older persons Am J Public Health 2007 97 486 492 10.2105/AJPH.2006.085837 17267713 PMC1805010

[B53] Van Dyck D Cardon G Deforche B Owen N De Bourdeaudhuij I Relationships between neighborhood walkability and adults’ physical activity: how important is residential self-selection? Health Place 2011 17 1011 1014 10.1016/j.healthplace.2011.05.005 21596613

[B54] Tilt JH Walking trips to parks: exploring demographic, environmental factors, and preferences for adults with children in the household Prev Med 2010 50 Suppl 1 S69 73 19744514 10.1016/j.ypmed.2009.07.026

[B55] Cohen DA Han B Derose KP Williamson S Marsh T Rudick J McKenzie TL Neighborhood poverty, park use, and park-based physical activity in a Southern California city Soc Sci Med 2012 75 2317 2325 10.1016/j.socscimed.2012.08.036 23010338 PMC3646794

[B56] Barnidge EK Radvanyi C Duggan K Motton F Wiggs I Baker EA Brownson RC Understanding and addressing barriers to implementation of environmental and policy interventions to support physical activity and healthy eating in rural communities J Rural Health 2013 29 97 105 10.1111/j.1748-0361.2012.00431.x 23289660 PMC4760835

[B57] Michimi A Wimberly MC Natural environments, obesity, and physical activity in nonmetropolitan areas of the United States J Rural Health 2012 28 398 407 10.1111/j.1748-0361.2012.00413.x 23083086

[B58] Pilling D Barrett P Text communication preferences of deaf people in the United Kingdom J Deaf Stud Deaf Educ 2008 13 92 103 17602163 10.1093/deafed/enm034

[B59] Hill JH Burge S Haring A Young RA Communication technology access, use, and preferences among primary care patients: from the Residency Research Network of Texas (RRNeT) J Am Board Fam Med 2012 25 625 634 10.3122/jabfm.2012.05.120043 22956698

[B60] Dunlop WL Beauchamp MR Birds of a feather stay active together: a case study of an all-male older adult exercise program J Aging Phys Act 2013 21 222 232 22899819 10.1123/japa.21.2.222

[B61] Fischer CS America Calling: A Social History of the Telephone to 1940 1940 Berkeley, CA: University of California Press 1992

[B62] Baron NS Always On: Language in an Online and Mobile World 2008 New York, NY: Oxford University Press

[B63] Hampton K Graham S Netville: community on and offline in a wired suburb The Cybercities Reader 2004 London: Routledge 260

[B64] Fowler JH Settle JE Christakis NA Correlated genotypes in friendship networks Proc Natl Acad Sci USA 2011 108 1993 1997 10.1073/pnas.1011687108 21245293 PMC3033315

[B65] Schaefer DR Simpkins SD Vest AE Price CD The contribution of extracurricular activities to adolescent friendships: new insights through social network analysis Dev Psychol 2011 47 1141 1152 21639618 10.1037/a0024091 PMC3134619

[B66] Hawe P Webster C Shiell A A glossary of terms for navigating the field of social network analysis J Epidemiol Commun H 2004 58 971 975 10.1136/jech.2003.014530 PMC1732636 15547054

[B67] Centola D The spread of behavior in an online social network experiment Science 2010 329 1194 1197 10.1126/science.1185231 20813952

[B68] Novembre J Johnson T Bryc K Kutalik Z Boyko AR Auton A Indap A King KS Bergmann S Nelson MR Genes mirror geography within Europe Nature 2008 456 98 101 10.1038/nature07331 18758442 PMC2735096

[B69] Boardman JD Domingue BW Fletcher JM How social and genetic factors predict friendship networks Proc Natl Acad Sci USA 2012 109 17377 17381 10.1073/pnas.1208975109 23045663 PMC3491494

[B70] McNeill LH Kreuter MW Subramanian SV Social environment and physical activity: a review of concepts and evidence Soc Sci Med 2006 63 1011 1022 10.1016/j.socscimed.2006.03.012 16650513

[B71] Christakis NA Fowler JH The spread of obesity in a large social network over 32 years N Engl J Med 2007 357 370 379 10.1056/NEJMsa066082 17652652

[B72] Christakis NA Fowler JH The collective dynamics of smoking in a large social network N Engl J Med 2008 358 2249 2258 10.1056/NEJMsa0706154 18499567 PMC2822344

[B73] Preciado P Snijders TAB Burk WJ Stattin H Kerr M Does proximity matter? Distance dependence of adolescent friendships Soc Networks 2012 34 18 31 10.1016/j.socnet.2011.01.002 PMC4268773 25530664

[B74] Rovniak LS Anderson ES Winett RA Stephens RS Social cognitive determinants of physical activity in young adults: a prospective structural equation analysis Ann Behav Med 2002 24 149 156 10.1207/S15324796ABM2402_12 12054320

[B75] Ogilvie D Foster CE Rothnie H Cavill N Hamilton V Fitzsimons CF Mutrie N Interventions to promote walking: systematic review BMJ 2007 334 1204 10.1136/bmj.39198.722720.BE 17540909 PMC1889976

[B76] Onnela JP Arbesman S Gonzalez MC Barabasi AL Christakis NA Geographic constraints on social network groups PLoS One 2011 6 e16939 10.1371/journal.pone.0016939 21483665 PMC3071679

[B77] Hayes RJ Moulton LH Cluster Randomised Trials 2009 Boca Raton, FL: Chapman & Hall

[B78] Plotnikoff RC Todosijczuk I Johnson ST Karunamuni N Canada’s physical activity guide: examining print-based material for motivating physical activity in the workplace J Health Commun 2012 17 432 442 10.1080/10810730.2011.626504 22206294

[B79] Skinner BF Science and human behavior 1953 New York: Macmillan

[B80] Cervero R Kockelman K Travel demand and the 3Ds: density, diversity, and design Transport Res D-Tr E 1997 2 199 219 10.1016/S1361-9209(97)00009-6

[B81] Frank LD Schmid TL Sallis JF Chapman J Saelens BE Linking objectively measured physical activity with objectively measured urban form - findings from SMARTRAQ American Journal of Preventive Medicine 2005 28 117 125 10.1016/j.amepre.2004.11.001 15694519

[B82] Duncan DT Aldstadt J Whalen J Melly SJ Gortmaker SL Validation of walk score for estimating neighborhood walkability: an analysis of four US metropolitan areas Int J Environ Res Public Health 2011 8 4160 4179 10.3390/ijerph8114160 22163200 PMC3228564

[B83] Saelens BE Sallis JF Black JB Chen D Neighborhood-based differences in physical activity: an environment scale evaluation Am J Public Health 2003 93 1552 1558 10.2105/AJPH.93.9.1552 12948979 PMC1448009

[B84] Sallis JF Johnson MF Calfas KJ Caparosa S Nichols JF Assessing perceived physical environmental variables that may influence physical activity Res Q Exerc Sport 1997 68 345 351 10.1080/02701367.1997.10608015 9421846

[B85] Neighborhood Quality of Life Surveyhttp://sallis.ucsd.edu/measures.html

[B86] Dunlop WL Beauchamp MR The relationship between intra-group age similarity and exercise adherence Am J Prev Med 2012 42 53 55 10.1016/j.amepre.2011.08.018 22176846

[B87] Sallis JF Grossman RM Pinski RB Patterson TL Nader PR The development of scales to measure social support for diet and exercise behaviors Prev Med 1987 16 825 836 10.1016/0091-7435(87)90022-3 3432232

[B88] Noble BJ Borg GAV Jacobs I Ceci R Kaiser P A category-ratio perceived exertion scale - relationship to blood and muscle lactates and heart-rate Med Sci Sport Exer 1983 15 523 528 6656563

[B89] Kendzierski D DeCarlo KJ Physical activity enjoyment scale: two validation studies Journal of Sport and Exercise Psychology 1991 13 50 64

[B90] Welk GJ Schaben JA Morrow JR Jr Reliability of accelerometry-based activity monitors: a generalizability study Med Sci Sports Exerc 2004 36 1637 1645 15354049

[B91] Sasaki JE John D Freedson PS Validation and comparison of ActiGraph activity monitors J Sci Med Sport 2011 14 411 416 10.1016/j.jsams.2011.04.003 21616714

[B92] Schneider PL Crouter SE Bassett DR Pedometer measures of free-living physical activity: comparison of 13 models Med Sci Sports Exerc 2004 36 331 335 10.1249/01.MSS.0000113486.60548.E9 14767259

[B93] Tudor-Locke C Ainsworth BE Thompson RW Matthews CE Comparison of pedometer and accelerometer measures of free-living physical activity Med Sci Sports Exerc 2002 34 2045 2051 10.1097/00005768-200212000-00027 12471314

[B94] Centers for Disease Control and Prevention National Health Interview Survey 1990 Washington, DC: US Dept of Health and Human Services, Public Health Service, National Center for Health Statistics

[B95] Craig CL Marshall AL Sjostrom M Bauman AE Booth ML Ainsworth BE Pratt M Ekelund U Yngve A Sallis JF Oja P International physical activity questionnaire: 12-country reliability and validity Med Sci Sports Exerc 2003 35 1381 1395 10.1249/01.MSS.0000078924.61453.FB 12900694

[B96] Kohl HW Blair SN Paffenbarger RS Jr Macera CA Kronenfeld JJ A mail survey of physical activity habits as related to measured physical fitness Am J Epidemiol 1988 127 1228 1239 3369421 10.1093/oxfordjournals.aje.a114915

[B97] American College of Sports Medicine ACSM’s Guidelines for Exercise Testing and Prescription 2010 8 Baltimore, MD: Lippincott Williams & Wilkins

[B98] Pickering TG Hall JE Appel LJ Falkner BE Graves JW Hill MN Jones DH Kurtz T Sheps SG Roccella EJ Recommendations for blood pressure measurement in humans: an AHA scientific statement from the council on high blood pressure research professional and public education subcommittee J Clin Hypertens (Greenwich) 2005 7 102 109 10.1111/j.1524-6175.2005.04377.x 15722655 PMC8109470

[B99] Buysse DJ Reynolds CF 3rd Monk TH Berman SR Kupfer DJ The Pittsburgh sleep quality index: a new instrument for psychiatric practice and research Psychiatry Res 1989 28 193 213 10.1016/0165-1781(89)90047-4 2748771

[B100] Steckler A Linnan L Process Evaluation for Public Health Interventions and Research 2002 San Francisco, CA: Jossey-Bass

[B101] Chan CB Ryan DA Tudor-Locke C Health benefits of a pedometer-based physical activity intervention in sedentary workers Prev Med 2004 39 1215 1222 10.1016/j.ypmed.2004.04.053 15539058

[B102] Croteau KA A preliminary study on the impact of a pedometer-based intervention on daily steps Am J Health Promot 2004 18 217 220 10.4278/0890-1171-18.3.217 14748310

[B103] Wing RR Jeffery RW Benefits of recruiting participants with friends and increasing social support for weight loss and maintenance J Consult Clin Psychol 1999 67 132 138 10028217 10.1037//0022-006x.67.1.132

[B104] Valente TW Network interventions Science 2012 337 49 53 10.1126/science.1217330 22767921

[B105] Ostrom E A general framework for analyzing sustainability of social-ecological systems Science 2009 325 419 422 10.1126/science.1172133 19628857

[B106] Mabry PL Olster DH Morgan GD Abrams DB Interdisciplinarity and systems science to improve population health: a view from the NIH Office of Behavioral and Social Sciences Research Am J Prev Med 2008 35 S211 224 10.1016/j.amepre.2008.05.018 18619402 PMC2587290

